# Influences on ambulance staff’s understandings and safeguarding of ethical values

**DOI:** 10.1177/09697330251344170

**Published:** 2025-05-26

**Authors:** Sara Björklund, Peter Hagell, Mats Holmberg, Petra Lilja Hagell

**Affiliations:** 4342 Kristianstad University; Region Blekinge; 4342 Kristianstad University; 4180 Linnaeus University; 6140 Region Sörmland; 8097 Uppsala University; 4342 Kristianstad University

**Keywords:** Ambulance, caring encounters, ethical values, othering, person-centered, stereotyping

## Abstract

**Background:** Ambulance staff face ethical demands to safeguard patient dignity and autonomy in situations where these values may be threatened. However, influences on how patients are understood can undermine this safeguarding, potentially impacting health outcomes. To address this, increased knowledge of these influences is needed as well as how they may form the ability to protect ethical values.

**Aim:** The aim was to explore ambulance staff’s view of what influences their understandings of encounters with persons living in stigmatized neighborhoods.

**Research Design:** Transcripts from semi-structured interviews with ambulance staff were analyzed with content analysis.

**Participant and Research Context:** Twenty-seven ambulance staff members were included from two different Swedish ambulance districts.

**Ethical Considerations:** The study was conducted based on the Declaration of Helsinki and approved by the Swedish Ethical Review Authority.

**Findings:** Six categories emerged; Individual values; Colleagues; Associated organizations; Societal information; Professional experiences; and Management.

**Conclusion:** Ambulance staff’s understandings are influenced in a multifaceted way that can produce processes of othering toward persons tied to specific neighborhoods, ethnicities, or cultures. This process endangers the safeguarding of dignity and autonomy due to understandings of the patient as of less value. However, critical reflection and exposure to diverse perspectives can counteract this and protect these ethical values.

## Introduction

Dignity and autonomy are fundamental ethical values in health care, closely linked to human rights as well as a person’s sense of self-worth or power. These ethical values can be vulnerable to threats, such as suffering, which may require support from Ambulance Services (AS). However, when staff take control, patients may feel a loss of dignity and autonomy, potentially worsening their suffering. This places ethical demands on the ambulance staff where a person-centered approach can help safeguard these ethical values by promoting trust and open communication. Yet, a risk of stereotyping has been detected in AS health care encounters within stigmatized neighborhoods, which may impair these patients’ dignity and autonomy. However, little is known about the influences contributing to this risk and how the ambulance staff’s ability to safeguard ethical values is affected. Therefore, this study aimed to explore ambulance staff’s view of what influences their understandings of encounters with persons living in stigmatized neighborhoods. The findings are followed by a discussion exploring how these influences may impair patients’ dignity and autonomy as well as form ambulance staff’s ability to safeguard these ethical values.

## Conceptual framework

The ethical values of dignity and autonomy can be seen as central to all forms of care but may also be viewed as closely related to human rights.^1^ Dignity can be explained as the inherent worth of every person and autonomy as self-determination which underpins human rights frameworks.^2^ These ethical values can be connected to how persons understand themselves since these ethical values can influence the sense of self-worth (feeling valuable) as well as self-esteem (feeling capable).^3^ Self-worth and self-esteem are in turn closely connected to the sense of power (the ability to act or influence) and capacity (the inner and outer resources to do so).^2,3^ Therefore, dignity and autonomy are of importance for acknowledging a person’s inherent worth as well as for understanding the vulnerability connected to these ethical values.

The vulnerability, connected to persons’ dignity and autonomy, derives from threats from oneself (physically or emotionally) or from the surroundings (relational or social) that may lead to a sense of decreased power and capacity.^4,5^ Such threats can originate in suffering where persons are unable to manage themselves or the situation that places their dignity and autonomy at risk.^6^ In such a situation, support from the AS may be needed to alleviate the suffering as well as restore dignity and autonomy. However, these encounters can also be perceived as a risk of losing power to the ambulance staff when they take over the managing of the situation, potentially deepening the patient’s suffering.^7^ This places ethical demands on the ambulance staff, whose approach must safeguard the patient’s dignity and autonomy to enable the encounter to be caring. A caring encounter can be described as a space based on mutuality where the patient is empowered to influence the care.^8^ A person-centered approach, where the patient is invited to engage on equal terms, may enable such an encounter.^9^ This can promote trust and open communication, allowing for a deeper understanding of the patient’s needs and beliefs, and facilitate the safeguarding of dignity as well as autonomy.^10^

However, the risk of ambulance staff being influenced by stereotypes hindering their ability to safeguard dignity and autonomy has been identified in health care encounters with persons living in stigmatized neighborhoods.^11^ Stereotyping denies persons their dignity and autonomy by not recognizing them as equals, often based on their appearance or where they live.^12^ When general stereotyping in society against specific residential areas occurs, stigmatization of neighborhoods may develop that affect opportunities in life for persons living there.^13^ Stereotyping can lead to patients’ unique needs not being acknowledged, resulting in inadequate or denied care.^14^ The persons’ dignity and autonomy are then at risk, and the encounter can develop into being non-caring with further suffering as a consequence.^15^ This can result in a restrained communication of needs, nonadherence to treatment, or reluctance to seek care in the future, which can negatively impact health.^16^ Thus, ambulance staff’s ability to resist stereotypes is essential for protecting dignity and autonomy and thereby ensuring both quality care as well as patient health. However, disparities in AS health care encounters have been detected in neighborhoods at risk of stigmatization.^17–19^ This may indicate that the risk of stereotyping among ambulance staff and disparities within the AS is related. Research in other fields has shown that neighborhood stigmatization can influence professionals’ understandings in a stereotyping way that may contribute to systematic disparities.^20^ However, despite these insights, there is limited knowledge about what underlies the risk of stereotyping as well as disparities within the AS and how it may influence safeguarding dignity and autonomy. Therefore, knowledge of influences on ambulance staff’s understandings of health care encounters with persons living in stigmatized neighborhoods and how it may affect the ability to safeguard ethical values is needed. Without such knowledge, efforts to ensure dignity and autonomy in AS health care encounters may overlook key structural or contextual influences counteracting the ability to safeguard these ethical values among ambulance staff.

## Aim

The aim was to explore ambulance staff’s view of what influences their understandings of encounters with persons living in stigmatized neighborhoods.

## Research design

The study had an explorative qualitative design where transcripts of interviews with ambulance staff collected in a previous study^11^ were analyzed using content analysis.^21^

### Study setting

In Sweden, 59 neighborhoods have been defined by the Police Authority as having criminality that impacts the local community as well as having socioeconomic deprivation. Since 2015, these neighborhoods have been published in a publicly available list involving approximately 5% of the Swedish population (i.e., about 500 000 persons).^22^ The neighborhoods included in the list vary in size and demographic characteristics, but in general they have a lower socioeconomic status and a larger proportion of persons born in another country or with parents born in another country than the rest of Sweden.^23^ These neighborhoods have been identified as exposed to stigmatization, and the publication of the Police Authority’s list may contribute to this with negative consequences such as stereotyping, social exclusion, and discrimination against its residents.^24,25^

This study was conducted within the Swedish AS in two ambulance districts that both have neighborhoods included in the Police Authority list.^22^ The ambulance districts are located in different parts of Sweden that together have about 80 000 assignments/year. The Swedish AS is regulated by the National Board of Health and Welfare^26^ and is part of the Swedish welfare system financed by income tax, but some districts charge a fee for the service. The state regions provide the AS and are responsible for the ambulance staff’s competence, their work environment, and that the ambulance care complies with regulations.^27^ The AS is responsible for emergency care in a prehospital setting. This includes advanced assessment, treatment, and triage, which can result in transport to the emergency department, to definitive care, to another health care facility or non-conveyance. The AS also collaborates with the police, the fire department, and other health care facilities when needed.^27^

The Swedish ambulance assignments are distributed by the nationwide Emergency Medical Communication Center (EMCC), which replies to emergency calls, prioritizes assignments, and collaborates with the police and fire department in dispatching services. In addition, several regions have their own communication center that distributes ambulance assignments when the EMCC has identified the call as a health care matter. When assignments are allocated to an ambulance unit, they typically contain information about the patient, location, and reason for contact.^28^ Ambulance units are advanced life support units that usually consist of two staff members where one must be licensed to administer drugs, which most commonly implies a Registered Nurse (RN). In Sweden, RNs have a 3-year university education with a bachelor’s degree in nursing science and may also have completed a 1-year specialist training leading to a master’s degree in nursing science.^26^ One of the staff members can also be an Emergency Medical Technician (EMT). EMTs have a high school diploma or 1 year of higher vocational training.^28^ Further details about the study setting are available elsewhere.^11^

### Participants and data collection

In a previous study exploring ambulance staff’s understandings of health care encounters with persons living in stigmatized neighborhoods,^11^ additional rich narratives emerged about influences that participants viewed as forming their understandings. Recognizing that knowledge about such influences is essential for a more comprehensive understanding of these encounters, we reanalyzed the full dataset with a new aim. Thereby, this study shifts the focus from examining how ambulance staff understands these encounters to exploring what influences those understandings, highlighting the underlying mechanisms that shape, rather than express, their understandings. The interview guide thus did not explicitly involve questions about what influenced their understandings (Table 1), but such narratives emerged spontaneously or in response to follow-up questions during the interviews (Table 2).

**Table 1.** Interview guide.

Interview guide
How did you perceive the health care encounter with this person?
What did you perceive could provide support during the health care encounter?
What did you perceive could cause problems or difficulties during the health care encounter?
Do you want to add something?
Example follow-up questions
Can you develop that? How do you mean? You said… can you tell me more about that?

**Table 2.** Examples of narration that include influences on how ambulance staff understand health care encounters with persons living in stigmatized neighborhoods.

Participant	21	19	20
Questions	*You told me before that you get information via the media. How do you think that can affect the health care encounter?*	*You also talked about a group analysis in the staff room, can you describe?*	*You also told me when you saw the address in the alarm text [from the EMCC], before you got to the patient you started thinking about things that had happened before. Can you tell me more about this?*
Answers (abbreviated)	Yes, but it builds, it forms our prejudices, definitely… Of course it’s media angles, of course they highlight all of this. They never show the man with a doctor’s ID… the media describes the violence and the shootings… when you hear about these crimes, it is once again the young, foreign-born man, in a disadvantage area who has committed them. You’re probably naive or stupid if you don’t get any prejudices about it or grow some kind of protection mechanism within you to be cautious.	It is a learning that is not always a healthy learning, learning also includes the attitude, …it is not always that those who are the loudest lecturers in the staff room are the sharpest or do the best work, but they still become role models for many who believe that this is the truth, this is how we should act, this is how we should see and who maybe even don’t always think that much or make their own observations.	Yes, last week, there were PMs distributed, or whatever they are called, of recent events and I remember the police had meetings with us then, about just this area… we talked about it on a daily basis, at every shift, at the beginning of every shift, we talked about this particular area …when you see that you are going there [in the received assignment] …it’s not like you want to go there exactly, I have to admit that, I would rather escape.

Purposeful sampling was used, and the inclusion criterion was to have worked a minimum of 2 years in the AS to ensure sufficient experience. Unit managers or the first author gave information about the study at workplace meetings. Interest in participation was collected by the unit managers and the first author asked about consent to participate in the study. The study included 27 ambulance staff (7 RNs, 15 RNs with specialist training, and 5 EMTs; median age, 42; 12 women; median years in AS, 7). Data were collected through individual semi-structured interviews. Definitions of stigmatized neighborhoods and health care encounters were provided, and the interviews followed an interview guide where participants were encouraged to talk freely about a health care encounter with a person living in a stigmatized neighborhood. Interviews were conducted through face-to-face meetings (*n* = 7), telephone (*n* = 3), or online (*n* = 17) and then transcribed verbatim. Detailed descriptions of the sample, participants, and data collection are available elsewhere.^11^

### Data analysis

The data were analyzed by following Krippendorff’s five steps of content analysis^21^ supported by the NVivo software program (version 14, 1533) in steps two and three: *1*. *(Unitizing)* Transcribed interviews were selected as data material since all authors considered them appropriate relative to the aim of this study. *2*. *(Sampling)* The complete transcripts were read by the first and last authors. Content that was relevant to the aim was extracted by the first author and then jointly discussed with all authors. *3*. *(Coding)* The extracted sections were broken down into smaller text units and labeled with a code describing the content, resulting in 408 codes. Similar codes were grouped into categories. All text units, codes, and categories were then read by the last author before the first and last authors discussed and reviewed the content. Units, codes, and categories were then reviewed by all authors and discussed jointly. *4*. *(Data languages)* The first and last authors text-closed the categories content, followed by joint discussions with all the authors and revision. *5*. *(Analytical constructs)* The first author then reanalyzed the data to find meanings of the categories content and expanded the category descriptions based on these findings. This was then presented to all authors who discussed it and revised it together.

### Ethical considerations

The study was approved by the Swedish Ethical Review Authority (Approval No. 2021-06744-01) and conducted in accordance with the Declaration of Helsinki. All participants received oral and written information about authors’ professions, the aim of the study as well as the procedure and that they could withdraw at any time. Written informed consent was obtained from all participants before the interviews, which were audio-recorded. The digital sound recordings were transcribed and coded, password-protected, and saved on an encrypted university server, and transcriptions were pseudonymized.

## Findings

**Figure 1.**
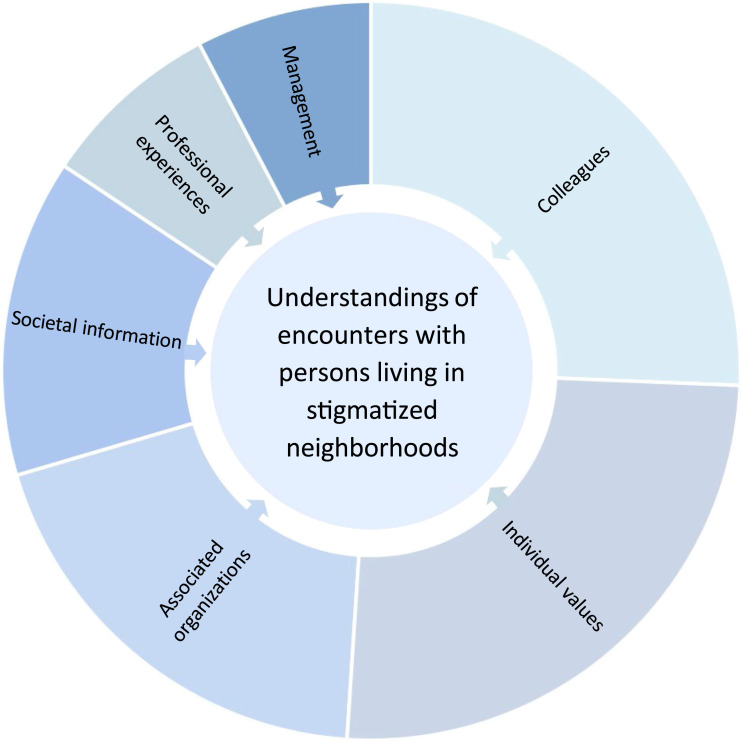
Categories influencing understandings presented in size depending on numbers of codes.

Six categories emerged: *Individual values* (138 codes); *Colleagues* (90 codes); *Associated organizations* (64 codes); *Societal information* (46 codes); *Professional experiences* (43 codes); and *Management* (27 codes). These are described separately below along with quotes and illustrated proportionally to the number of codes in Figure 1.

### Individual values

Cultural traditions, upbringing, and social standards were described by the ambulance staff as generating individual values. These values were viewed as forming personal qualities involving perspectives on humanity, helpfulness, and openness. These individual values were described as either reinforcing or challenging personal as well as broader understandings among themselves, at their workplace or in society as a whole.

“*I think it’s just how you are as a person, your view of people and how you treat people and how you were brought up. I don’t think any nursing science or anything like that can help you encounter people well if you, like, if you’re not like that as a human being yourself*.” (Participant 13)

The ability to be aware of different understandings and to critically appraise them was described as a necessity to avoid being influenced by preconceived notions when encountering patients. The ambulance staff described that acknowledging and questioning understandings about specific neighborhoods, ethnicities, or cultures generates a resistance toward the temptation to categorize patients based on stereotypes. This ability was viewed as being cultivated through relationships where understandings are challenged, leading to the development of new insights.

### Colleagues

When an ambulance unit receives an assignment, colleagues were described as jointly expressing opinions about the causes of symptoms and what type of care was required. This collective meaning-making process was viewed as forming a shared understanding which shapes expectations and conclusions before encountering the patient. This was viewed as shaping a general understanding of how to approach persons living in specific neighborhoods despite differences in the assignment or in needs. This generalizing approach potentially hinders the ambulance staff’s ability to recognize and respond to unique needs but also forcing them to muster enough courage to develop their own approach.

“*If he is super sure and says like this: I promise you this, then it colors my picture of the situation, and you learn from your experienced colleagues. I then think that of course he has seen this sort of thing, he certainly knows what he is talking about! And you are probably influenced by your colleagues, especially if you are new. If the experienced colleagues behave like this and spread these stereotypes, then it forms some kind of culture in the workplace that is easily transferred to new recruits*.” (Participant 14)

Colleagues’ stories about their previous experiences were tolerated even when exaggerated. Stories involving patients living in specific neighborhoods were not described in terms of individual variations but through generalized assumptions tied to specific ethnicities or cultures. These stories were often considered harsh as well as judgmental, contributing to an “us-and-them” where the patient was viewed as fundamentally different or even inferior. These stories were viewed as forming a culture with a collective understanding and a resistance to alternative viewpoints, influencing how patients are understood. This made it difficult to express one’s own perspective when it conflicted with the prevailing understanding and was seen as generating a struggle to avoid being influenced by general understandings occurring in the workplace.

### Associated organizations

The ambulance staff’s understanding of persons living in specific neighborhoods was viewed as influenced by the perceived attitudes of staff within the EMCC and the emergency department. This influence was viewed as reinforcing existing preconceptions or creating an internal struggle, where one had to actively resist being blinded by stereotypes that were strengthened through these shared attitudes. The ambulance staff described how this influence could shape preconceived notions about patients’ needs and how they should be approached.

“*Sometimes in the alarm text, the EMCC staffs´ perceptions shine through. Even if they write briefly and with words like*… *it often shines through if they have had a conversation that has been*…*difficult due to language barriers and it may have been noisy in the background and maybe there was also irritation. You can almost see it from the alarm text. And then, somewhere, something happens in your brain and in your body, and you also prepare for that*.” (Participant 6)

The categorization of neighborhoods as being “high-risk” was viewed as influenced by official lists of residential areas defined by the Polis Authority, alongside information about local areas gained through collaborations with local law enforcement. This contributed to a preemptive understanding and to a narrative of danger associated with both the place and its residents, which framed encounters through a lens of suspicion. This influence was viewed as shaping a distance between the ambulance staff who must protect themselves and the patient who is understood as a potential source of risk. The distance reduced persons, living in neighborhoods seen as high risk, to be understood as representatives of a problematic area, overshadowing their unique needs.

### Societal information

Information received through media or community hearsay was described as shaping broad, generalized understandings not only of specific neighborhoods but also of persons associated with specific ethnicities or cultures. These narratives were often considered problem-focused, frequently linking issues to persons with a foreign background. This was viewed as reinforcing broader stereotypes linked to ethnicities or cultures, contributing to a perception of “us-and-them” that extended beyond specific neighborhoods.

“*When you hear about these crimes, it is once again the young, foreign-born man, in a disadvantaged area who has committed them. You’re probably naive or stupid if you don’t get any prejudices about it or grow some kind of protection mechanism within you to be cautious*.” (Participant 21)

The understanding of threats against ambulance staff during assignments was viewed as influenced by media and community narratives about specific neighborhoods. These narratives, often centered around crime or violence, were considered influential regardless of whether the incidents involved the AS or occurred locally or elsewhere in Sweden. The spread of such narratives was seen as contributing to a generalized understanding of specific neighborhoods, framing them as inherently dangerous and forming a distancing process from the persons living there. However, when personal understanding conflicted with these narratives, the ambulance staff expressed resistance. They described understandings of injustice toward those targeted by these narratives as well as a responsibility to avoid being blinded by these influences.

### Professional experiences

The ambulance staff described a tendency to form generalized understandings based on their professional experiences with patients in specific neighborhoods. This influence was seen as potentially hindering their ability to adapt care to unique needs, raising a risk of making assumptions when providing care. The ambulance staff noted that due to this tendency to form generalized understandings, they had to consciously work harder to prevent them from influencing their approach in the encounter with the patient.

“*All these encounters you’ve had, some you completely forget but most of them you still kind of recall a little: oh, I have been in that building before, doesn’t an old lady live there? If you’ve been there before and you get that address it’s like: isn’t that where he or she lives, do you remember when we were there?*… *And it is similar when it comes to these neighborhoods, but the difference is that you generalize a bit more*… *It is a special neighborhood, and they are sweeping generalizations*.” (Participant 21)

The influencing professional experience involved health care encounters in neighborhoods considered generally singled out as different, as well as encounters in other residential areas where the patients were associated with specific characteristics (e.g., ethnicities, cultures, drug use, and violence). This influence was formed by interactions with “those” perceived as unfamiliar and different from oneself. This was viewed as resulting in an understanding where the ambulance staff and the person in need of care were seen as belonging to separate groups in society with little in common. However, the ambulance staff also described understandings of recognition or togetherness despite differences in backgrounds. Such understandings were viewed as influenced by the own capability to see beyond generalizations and stereotypes, which was facilitated by reflection of own viewpoints as well as external influences.

### Management

The lack of management focus on reflection after assignments was viewed as preventing awareness of prejudices as well as behaviors. This was seen as reinforcing unchallenged understandings, where assumptions were accepted as truths without critical reflection. This was described as a process where unchallenged viewpoints shape how ambulance staff understand and interacted with patients considered as not belonging to their own group. When working with a colleague who showed interest in reflecting on understandings and experiences, this process was instead transformative and enhanced the ability to gain new insights.

“*To be given the opportunity [by the management] to see things in different ways and discuss a thing and talk about it*… *just like I do now, I see things in a different way. Meanwhile, if I hadn’t talked about it, it would have just been the experience and the perception that I had right after and it would have remained the same, as it were. And it is obvious that it would have affected a similar health care encounter*.” (Participant 6)

The management’s strong emphasis on safety strategies was seen as influencing a collective understanding of threats to personal safety when encountering patients in specific neighborhoods. This focus on safety contributed to an understanding of specific neighborhoods as inherently dangerous and that ambulance staff should maintain a distance from those living there for their own protection. The distance was seen as hindering care from being responsive toward patients’ needs. The ambulance staff also described that such distance formed an understanding of guilt as they struggled with the inability to connect on a human level or provide empathetic care.

## Discussion

The findings indicate that the ambulance staff’s understandings are influenced in a multifaceted way where there is a risk of processes of othering which may impair their ability to safeguard dignity and autonomy. The process of othering defines specific groups as fundamentally different from the perceived norm, thus connecting them to stereotypes.^29^ This process undermines the safeguarding of dignity and autonomy due to understandings of patients as deviant, where they might be disrespected by not having their needs acknowledged.^30,31^ The findings indicate that influences by *Colleagues*, *Associated organizations*, *Societal information*, *Professional experiences*, and *Management* can generate such a process of othering toward patients tied to specific neighborhood, ethnicity, or culture. This may contribute to explaining the increased risk of stereotyping within the AS toward patients in stigmatized neighborhoods.^11^ Ambulance staff described how a prevailing group culture, often resistant to differing viewpoints, contributed to generalized understandings. Such understandings could also be further reinforced by interactions with other involved organizations, shaping a collective perspective. This reflects a divide between “us” (the in-group) and “them” (the out-group) formed by the othering process. This categorization is not only about cultural or social differences but also reflects power dynamics, where the dominant group defines itself as superior while marginalizing the other group.^29^ Broad influences spanning across collaborating organizations may help explain how structural disparities arise within the AS and the wider emergency care sector, particularly impacting patients linked to specific neighborhoods, ethnicities, or cultures.^32^ These understandings undermine the safeguarding of dignity and autonomy, as it may result in patients being seen as less competent and excluding them from meaningful involvement in decisions about their own care.^29,33^ Such experiences can negatively impact health by influencing care-seeking behaviors or leading to further suffering due to feelings of not belonging.^34^ Thus, influences risking an othering process that impair the safeguarding of dignity and autonomy may compromise health and care outcomes.

The findings suggest a risk of distancing patients in specific neighborhoods, influenced by perceived threats to personal safety shaped by societal narratives and official classifications through influences by *Associated organizations*, *Societal information*, and *Management*. Similar influences have been observed in other professions, revealing a risk of disparities that suggest a continued reproduction of stigmatization toward specific neighborhoods.^20^ The reproduction of stigmatization through distancing can obscure its deeper implications, as the exclusion involved conceals the lived experiences and consequences faced by those who are seen as not belonging.^12,29^ This can seriously undermine dignity and autonomy, as it may prevent recognition of the shared humanity.^1,29^ When the shared humanity is not recognized, patients risk being objectified, which can affect both care and communication and lead to experiences of not having the legitimacy to influence one’s own care.^33^ Undermining of ethical values due to stigmatization may therefore influence more than just the delivery of care; it may also affect the person’s own understanding of their right to equitable care.

The findings indicated that influences by *Colleagues*, *Societal information*, and *Professional experiences* may deepen the othering process by shaping broad generalizations that extended beyond specific neighborhoods to include entire ethnic or cultural groups. This may suggest that the process of othering is not inherently tied to specific neighborhoods. Instead, the risk of othering can be traced in societal structures with a historical origin that reproduce stereotyping and imbalanced power relations in society.^12,29^ Such othering counteracts the safeguarding of ethical values through structures fixed in laws, policies, and institutions such as the health care sector.^35^ This can deepen the understanding of disparities existing within the entire health care sector that affect both possibilities for care and health depending on ethnicity and culture.^36^ However, the findings also suggest that influences from *Individual values* may strengthen the capacity to safeguard patient dignity and autonomy through the ability to reflect upon one’s own understandings and their influences. Such an ability can be described as self-awareness, an active recognition of own preconceived notions and how these may shape both the understanding of the patient and the own behavior.^37^ Awareness of one’s own understandings can facilitate the possibility to set them aside, allowing the patient’s boundaries for dignity and autonomy to become visible, and thereby enabling the safeguarding of these ethical values.^33^ According to the findings, this ability seems to potentially safeguard ethical values even in the face of negative structures and influences such as through *Societal information* and *Professional experiences*. However, a struggle was described, even though this ability might exist, when generalized influences were experienced by *Colleagues* and *Associated organizations*. To facilitate and develop this ability, the opportunity for reflection with colleges as well as engaging with alternative perspectives was considered important. Therefore, it is important to actively facilitate opportunities for reflection and exposure to alternative perspectives among ambulance staff. This can help counteract the effects of othering, support the safeguarding of patients’ dignity and autonomy, and reduce potential negative consequences for health.

## Implications for further research

While our findings imply that reflection is of importance to counteract othering as well as to safeguard dignity and autonomy, they also point to the need for better understanding of other perspectives. However, there is a lack of studies exploring the perspectives of persons at risk of othering during encounters with ambulance staff in stigmatized neighborhoods. To address this gap, research that deepens the understanding of patients’ experiences and viewpoints in these contexts is needed.

## Strength and limitations

Since understandings are often formed unconsciously, the ambulance staff’s own capability to detect influences may be limited. However, despite the interviews’ primary focus, as not involving questions about influences, rich narratives emerged on what participants perceived influenced their health care encounters. This may indicate that un-reflected information such as unconscious influences were revealed, and thus increasing the study’s *credibility*.^38^ Still, influences of understanding cannot be stated in terms of cause and effect since the study has adopted a qualitative method. However, this study provides valuable insights despite the challenges of studying this field and is of importance in the preventive work counteracting discrimination based on stereotyping.

## Conclusion

Ambulance staff’s understandings are formed by various influences that can contribute to or perpetuate processes of othering toward patients tied to specific neighborhoods, ethnicities, or cultures. This can threaten the safeguarding of patients’ dignity and autonomy leading to further suffering and consequences for health. These insights may contribute to an understanding of the risk for stereotyping and disparities identified in AS health care encounters with persons living in stigmatized neighborhoods. However, the findings indicate that critical reflection and opportunities to be challenged by diverse perspectives can help counteract this othering process, ultimately safeguarding ethical values despite the presence of negative influences.

## Data Availability

The datasets generated during and/or analyzed during the current study are not publicly available due to requirement for confidentiality of the participant by the Ethical Review Authority but are available from the corresponding author on reasonable request.*

## References

[1] Gallagher A . Dignity and respect for dignity--two key health professional values: implications for nursing practice. Nurs Ethics 2004; 11: 587–599. DOI: 10.1191/0969733004ne744oa. 15597939

[2] Van Brussel L . Autonomy and dignity: a discussion on contingency and dominance. Health Care Anal 2014; 22: 174–191. DOI: 10.1007/s10728-012-0217-0. 22760175

[3] Stoljar N Mackenzie C . Relational autonomy in feminist bioethics. In: Rogers LJS AW Carter MS Entwistle V , et al. (eds) The Routledge Handbook of Feminist Bioethics. New York, NY: Routledge, 2023.

[4] Clark B Preto N . Exploring the concept of vulnerability in health care. CMAJ (Can Med Assoc J) 2018; 190: E308–E309. DOI: 10.1503/cmaj.180242. 29555859 PMC5860890

[5] Jones DA . Human dignity in healthcare: a virtue ethics approach. N Bioeth 2015; 21: 87–97. DOI: 10.1179/2050287715z.00000000059. 29384347

[6] Rodgers BL Cowles KV . A conceptual foundation for human suffering in nursing care and research. J Adv Nurs 1997; 25: 1048–1053. DOI: 10.1046/j.1365-2648.1997.19970251048.x. 9147211

[7] Holmberg M Forslund K Wahlberg AC , et al. To surrender in dependence of another: the relationship with the ambulance clinicians as experienced by patients. Scand J Caring Sci 2014; 28: 544–551. DOI: 10.1111/scs.12079. 24067194

[8] Holopainen G Kasén A Nyström L . The space of togetherness--a caring encounter. Scand J Caring Sci 2014; 28: 186–192. DOI: 10.1111/j.1471-6712.2012.01090.x. 23039849

[9] Tomaselli G Buttigieg SC Rosano A , et al. Person-centered care from a relational ethics perspective for the delivery of high quality and safe healthcare: a scoping review. Front Public Health 2020; 8: 44. DOI: 10.3389/fpubh.2020.00044. 32211362 PMC7067745

[10] Delmar C . The interplay between autonomy and dignity: summarizing patients voices. Med Health Care Philos 2013; 16: 975–981. DOI: 10.1007/s11019-012-9416-6. 22623342

[11] Bjorklund S Lilja Hagell P Hagell P , et al. Ambulance staff's ways of understanding health care encounters in stigmatized neighborhoods - a phenomenographic study. Int Emerg Nurs 2024; 74: 101451. DOI: 10.1016/j.ienj.2024.101451. 38663203

[12] Andersen MM Varga S Folker AP . On the definition of stigma. J Eval Clin Pract 2022; 28: 847–853. DOI: 10.1111/jep.13684. 35462457 PMC9790447

[13] Larsen TS Delica KN . The production of territorial stigmatisation. City 2019; 23: 540–563. DOI: 10.1080/13604813.2019.1682865.

[14] Hall WJ Chapman MV Lee KM , et al. Implicit racial/ethnic bias among health care professionals and its influence on health care outcomes: a systematic review. Am J Publ Health 2015; 105: e60–e76. DOI: 10.2105/AJPH.2015.302903. PMC4638275 26469668

[15] Nystrom M Dahlberg K Carlsson G . Non-caring encounters at an emergency care unit--a life-world hermeneutic analysis of an efficiency-driven organization. Int J Nurs Stud 2003; 40: 761–769. DOI: 10.1016/s0020-7489(03)00053-1. 12965167

[16] Hamed S Bradby H Ahlberg BM , et al. Racism in healthcare: a scoping review. BMC Public Health 2022; 22: 988. DOI: 10.1186/s12889-022-13122-y. 35578322 PMC9112453

[17] Niklasson A Herlitz J Jood K . Socioeconomic disparities in prehospital stroke care. Scand J Trauma Resuscitation Emerg Med 2019; 27: 53. DOI: 10.1186/s13049-019-0630-6. PMC6498576 31046804

[18] Dawson LP Andrew E Nehme Z , et al. Association of socioeconomic status with outcomes and care quality in patients presenting with undifferentiated chest pain in the setting of universal health care coverage. J Am Heart Assoc 2022; 11: e024923. DOI: 10.1161/JAHA.121.024923. 35322681 PMC9075482

[19] Hsia RY Huang D Mann NC , et al. A US national study of the association between income and ambulance response time in cardiac arrest. JAMA Netw Open 2018; 1: e185202. DOI: 10.1001/jamanetworkopen.2018.5202. 30646394 PMC6324393

[20] Braga AA Brunson RK Drakulich K . Race, place and effective policing. Annu Rev Sociol 2019; 45: 535–555. DOI: 10.1146/annurev-soc-073018-022541.

[21] Krippendorff K . Content analysis-an introduction to its methodology. Thousand Oaks, CA: Sage Publication, 2019.

[22] Polismyndigheten (The Swedish Police Authority) . Lägesbild över utsatta områden. Report no. A658.585/2023. Stockholm, Sweden: Polismyndigheten (The Swedish Police Authority), 2023.

[23] The Global Village . Fakta för förändring- en rapport om Sveriges 61 utsatta områden. https://jarvaveckan.se/wp-content/uploads/2023/05/Fakta-for-forandring-%E2%80%93-Sveriges-61-utsatta-omraden.pdf (2023, accessed 2025-02-16).

[24] Fell T Rydenstam T Geleta BB , et al. Citizen science in Sweden’s stigmatized neighborhoods. Sustainability 2021; 13(18): 10205. DOI: 10.3390/su131810205.

[25] Roelofs K . Concealed stigmatizaion. Lund, Sweden: Lunds University, 2024.

[26] Socialstyrelsen (National Board of Health and Welfare) . Socialstyrelsens föreskrifter om ambulanssjukvård m.m. Stockholm, Sweden: Socialstyrelsen (National board of Health and Welfare), 2009, p. 10.

[27] Socialstyrelsen (National Board of Health and Welfare) . Sveriges prehospitalta akutsjukvård- nulägesbild, bedömning och utvecklingsförslag. Report no. 2023-2-8337. Stockholm, Sweden: Socialstyrelsen (National board of Health and Welfare), 2023.

[28] Lindstrom V Borm K Kurland L . Prehospital care in Sweden. Notfall Rettungsmed 2015; 18: 107–109. DOI: 10.1007/s10049-015-1989-1.

[29] Roberts MLA Schiavenato M . Othering in the nursing context: a concept analysis. Nurs Open 2017; 4: 174–181. DOI: 10.1002/nop2.82. 28694982 PMC5500989

[30] Canales MK . Othering: toward an understanding of difference. ANS Adv Nurs Sci 2000; 22: 16–31. DOI: 10.1097/00012272-200006000-00003. 10852666

[31] Lin YP Watson R Tsai YF . Dignity in care in the clinical setting: a narrative review. Nurs Ethics 2013; 20: 168–177. DOI: 10.1177/0969733012458609. 23131700

[32] Hsia RY Zagorov S . Structural discrimination in emergency care: how a sick system affects us all. Méd Sur 2022; 3: 98–103. DOI: 10.1016/j.medj.2022.01.006. PMC8880827 35224522

[33] Stephen Ekpenyong M Nyashanu M Ossey-Nweze C , et al. Exploring the perceptions of dignity among patients and nurses in hospital and community settings: an integrative review. J Res Nurs 2021; 26: 517–537. DOI: 10.1177/1744987121997890. 35265158 PMC8899300

[34] Akbulut N Razum O . Why othering should be considered in research on health inequalities: theoretical perspectives and research needs. SSM Popul Health 2022; 20: 101286. DOI: 10.1016/j.ssmph.2022.101286. 36406107 PMC9672483

[35] Elias A Paradies Y . The costs of institutional racism and its ethical implications for healthcare. J bioeth Inq 2021; 18: 45–58. DOI: 10.1007/s11673-020-10073-0. 33387263 PMC7778398

[36] Webster CS Taylor S Thomas C , et al. Social bias, discrimination and inequity in healthcare: mechanisms, implications and recommendations. BJA Educ 2022; 22: 131–137. DOI: 10.1016/j.bjae.2021.11.011. 35531078 PMC9073302

[37] Younas A Rasheed SP Sundus A , et al. Nurses’ perspectives of self-awareness in nursing practice: a descriptive qualitative study. Nurs Health Sci 2020; 22: 398–405. DOI: 10.1111/nhs.12671. 31837204

[38] Stahl NA King JR . Expanding approaches for research: understanding and using trustworthiness in qualitative research. J Dev Educ 2020; 44: 26–28.

